# Long-term progression-free survival in an advanced lung adenocarcinoma patient harboring EZR-ROS1 rearrangement: a case report

**DOI:** 10.1186/s12890-018-0585-9

**Published:** 2018-01-23

**Authors:** Liang Dong, Jingwen Xia, Jing Zhang, Yuanyuan Zhang, Ning Zhu, Peng Zhang, Youzhi Zhang, Xiujuan Zhang, Shengqing Li

**Affiliations:** Department of Pulmonary and Critical Care Medicine, Huashan Hospital, Fudan University, #12, Urumqi middle Road, Shanghai, 200040 China

**Keywords:** Lung adenocarcinoma, ROS1 rearrangement, Pemetrexed, Crizotinib, Lorlatinib

## Abstract

**Background:**

Crizotinib is recommended as first-line therapy in ROS1-driven lung adenocarcinoma. However, the optimal first-line therapy for this subgroup of lung cancer is controversial according to the available clinical data.

**Case presentation:**

Here, we describe a 57-year-old man who was diagnosed with stage IIIB lung adenocarcinoma and EGFR/KRAS/ALK-negative tumors. The patient received six cycles of pemetrexed plus cisplatin as first-line therapy and then pemetrexed as maintenance treatment, with a progression-free survival (PFS) of 42 months. The patient relapsed and underwent re-biopsy. EZR-ROS1 fusion mutation was detected by next-generation sequencing (NGS). The patient was prescribed crizotinib as second-line therapy and achieved a PFS of 6 months. After disease progression, lorlatinib was administered as third-line therapy, with a favorable response.

**Conclusions:**

Prolonged PFS in patients receiving pemetrexed chemotherapy might be related to the EZR-ROS1 fusion mutation. Lorlatinib is an optimal choice in patients showing crizotinib resistance.

## Background

Prolonging the overall survival (OS) of advanced lung cancer patients remains a challenge. The advent of targeted therapeutic approaches led to the classification of NSCLC into subgroups according to factors such as histology and the molecular makeup of the tumor. C-ros oncogene 1 (ROS1) rearrangements are detected in approximately 1–2% of patients with NSCLC [1, 2]. ROS1 is a receptor tyrosine kinase (RTK) related to the anaplastic lymphomakinase/lymphocyte-specific protein tyrosine kinase (ALK/LTK) and insulin receptor (INSR) RTK families [3, 4]. Preclinical and clinical data support the efficacy of tyrosine kinase inhibitors (TKIs) against these receptors, such as crizotinib (first generation) [5, 6], ceritinib (second generation) [7], and lorlatinib (third generation) [8, 9], in ROS1-positive NSCLC patients. However, a case series study reported prolonged PFS with pemetrexed as first-line and maintenance therapy in patients with ROS1-driven lung adenocarcinoma [10], indicating that patients in this subgroup may be optimal candidates for pemetrexed chemotherapy.

We herein report a case of advanced lung adenocarcinoma with EZR-ROS1 rearrangement treated by first-line pemetrexed/cisplatin and then pemetrexed mono-drug for maintenance therapy. After progression, crizotinib was used as second-line treatment, and lorlatinib as third-line treatment. The patient showed an excellent response and achieved long-term progression-free survival (PFS).

## Case presentation

**Fig. 1:**
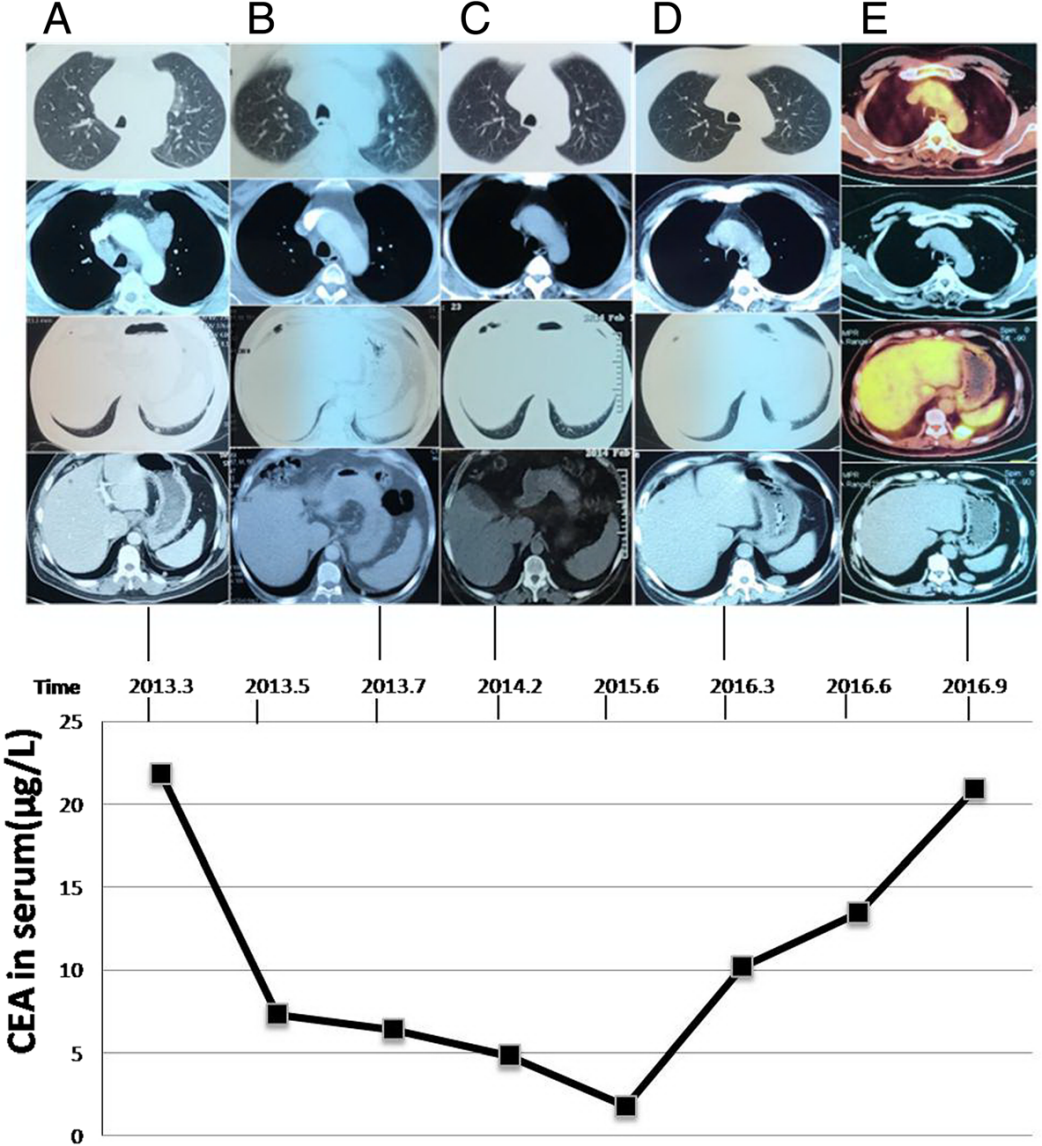
Follow-up schematic diagram between March 2013 and September 2016. The top panel shows a series of CT scans (columns **a**–**d**) and a PET/CT scan (column **e**) at different time points as indicated. The upper two rows of images of the lung window and mediastinal window depict the changes of enlarged mediastinal lymph nodes, which shrunk after six cycles of pemetrexed plus cisplatin chemotherapy and nine cycles of pemetrexed maintenance (columns **a**–**c**). They remained stable after four cycles of pemetrexed plus cisplatin chemotherapy (columns **d**–**e**). The lower two rows of images of the lung window and mediastinal window depict the changes of the primary lesion as a 9 × 11 mm nodule (column **a**) in the left lung lower lobe. The lesion decreased in size after six cycles of pemetrexed plus cisplatin chemotherapy and nine cycles of pemetrexed maintenance (columns **a**–**c**), but relapsed with an enlarged 31 × 15 mm nodule in March 2016 (column **d**). Four cycles of pemetrexed plus cisplatin chemotherapy were unsuccessful in controlling the lesion, and a growing nodule of 32 × 17 mm was detected (column **e**). The bottom panel depicts the follow-up changes of serum CEA levels, which markedly decreased after chemotherapy, but increased in March 2016 after disease progression. CT, computed tomography; PET, positron emission tomography; CEA, carcinoembryonic antigen

A 57-year-old man with a 20-pack-year smoking history presented to the hospital in March 2013 with a persistent cough for 2 months and a palpable right cervical mass for 4 days. Enhanced computed tomography (CT) showed a 9 × 11 mm nodule in the lower lobe of the left lung and multiple enlarged lymph nodes (Fig. 1a). The serum levels of carcinoembryonic antigen (CEA) were 21.86 μg/L (Fig. 1, lower panel). A cervical lymph node biopsy confirmed the diagnosis of lung adenocarcinoma. The biopsy sample was genotyped negative for EGFR and KRAS mutations using an amplification refractory mutation system (ARMS)-polymerase chain reaction (PCR) method and negative for ALK rearrangement by fluorescent in situ hybridization (FISH). At that time, ROS1 rearrangement was not detected because of the lack of a detection method and no targeted drug available in clinical practice. The patient’s clinical stage was determined as cT1aN3M0 (stage IIIB). Accordingly, he received first-line chemotherapy with six cycles of pemetrexed (500 mg/m^2^) and cisplatin (75 mg/m^2^), achieving a partial response (Fig. 1b). Maintenance therapy consisted of nine cycles of pemetrexed (500 mg/m^2^) every month (Fig. 1c). Assessment of CEA levels and a lung CT scan were performed every 2–3 months during the follow-up period. In March 2016, the patient showed a marked increase in serum CEA levels from 1.78 μg/L to 10.21 μg/L, and a CT scan showed pulmonary disease progression with an enlarged 31 × 15 mm nodule (Fig. 1d). The patient was treated with four cycles of pemetrexed (500 mg/m^2^) and cisplatin (75 mg/m^2^) at a local hospital. At last, the pemetrexed-based regimen for this patient resulted in a PFS of 42 months.

**Fig. 2:**
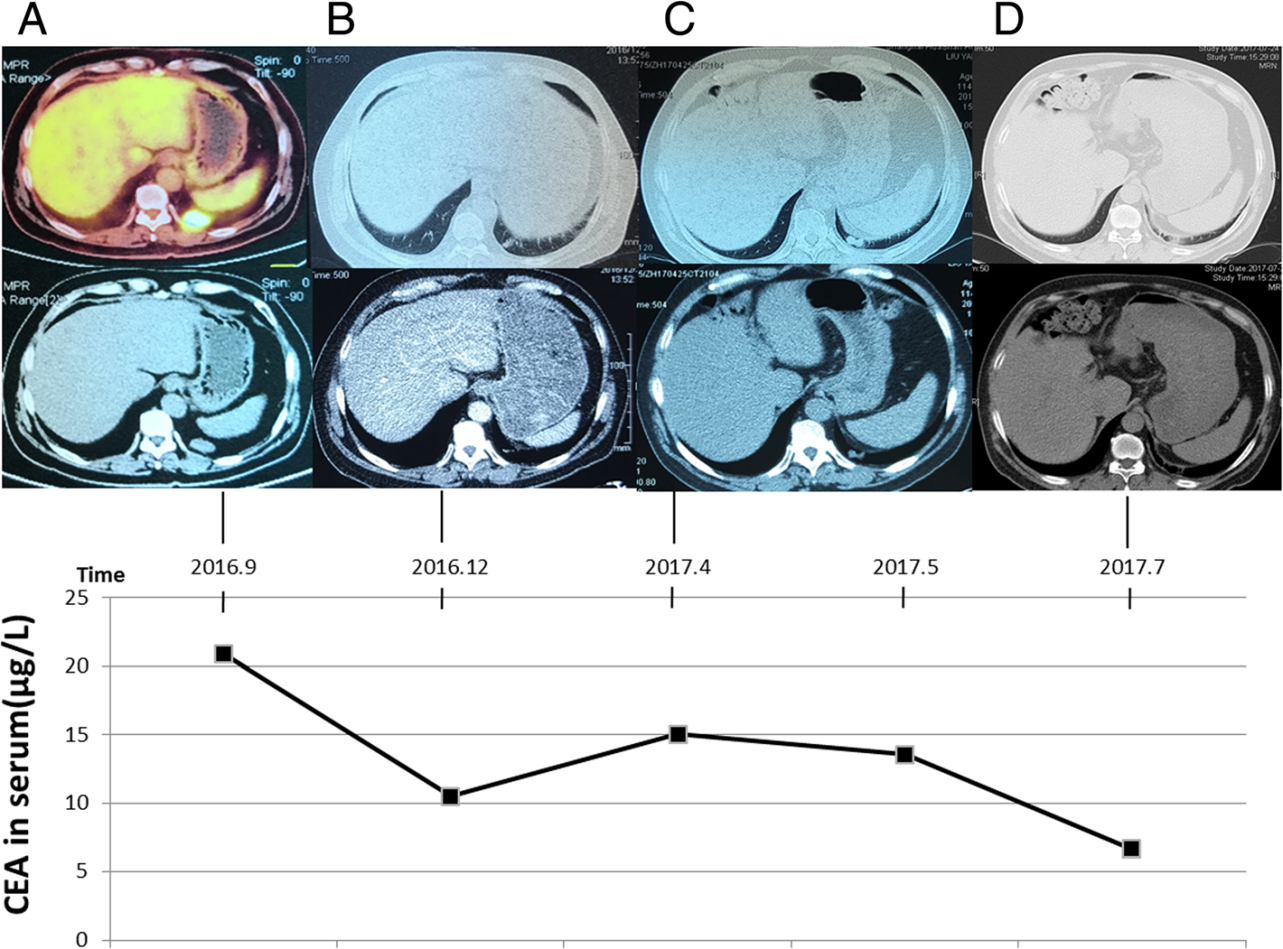
Follow-up schematic diagram between September 2016 and July 2017. The top panel shows a PET/CT scan (column **a**) and a series of CT scans of the lung window and mediastinal window (columns **b**–**d**) of the primary lesion, which decreased in size after crizotinib treatment targeting the EZR-ROS1 fusion protein (columns **a** and **b**). However, relapse was detected in April 2017 (column **c**), which was controlled with lorlatinib until now (column **d**). The bottom panel depicts the follow-up changes of serum CEA levels, which markedly decreased after crizotinib treatment, and increased in April 2017 after disease progression, showing a decreasing trend after lorlatinib treatment. CT, computed tomography; PET, positron emission tomography; CEA, carcinoembryonic antigen

The patient was referred to our hospital because of disease progression. Following admission, a positron emission tomography (PET)/CT scan revealed a 32 × 17 mm nodule in the left lower lobe with intense uptake of 18F–fluorodeoxyglucose and multiple hypermetabolic lymph nodes (Figs. 1e and 2a). To understand the histological and molecular evolution of cancer tissues, a secondary biopsy was performed by endobronchial ultrasonography with a guide sheath (EBUS-GS), which confirmed the diagnosis of adenocarcinoma by immunohistochemical staining. Target-capture sequencing on an Illumina platform was performed in a laboratory certified by the College of American Pathologists using a next-generation sequencing (NGS) method across a 123 gene panel. Tumor samples showed activating mutations in EZR-ROS1, as well as the concomitant alterations NQO1(P187S), TYMS (− 6 bp non-frame shift deletion), UGT1A1(G71R), XRCC1(Q339R), CYP2D6(P34S), DPYD (I543V), and MTHFR (A222V). The tumor was therefore identified as ROS1-driven lung cancer, and the patient was immediately started on second-line therapy with crizotinib (250 mg/twice daily), which targets ROS1 rearrangements. Within 3 months of crizotinib treatment, the patient had achieved excellent radiological partial remission (Fig. 2b) according to the Response Evaluation Criteria in Solid Tumors version 1.1 (RECIST*v*1.1). In April 2017, serum CEA levels were increased, and a lung CT scan showed an enlarged nodule (Fig. 2c). The PFS on crizotinib was determined as 6 months. Lorlatinib, a third-generation macrocyclic ALK/ROS1-TKI (100 mg/once daily), was used as third-line therapy after obtaining consent from the patient. After 3 months, the patient achieved a favorable response (Fig. 2d), which was maintained at the time of publication.

Written informed consent was obtained from the patient for the publication of this case report.

## Discussion and conclusions

Crizotinib is approved by the US Food and Drug Administration (FDA) for the first-line treatment of NSCLC patients with ROS1 rearrangements with a median PFS of at least 19.2 months [6, 11]. Pemetrexed-based regimens are recommended as first-line and maintenance therapy for advanced lung adenocarcinoma, particularly in patients without driven mutations [12–14]. However, pemetrexed shows superior efficacy as first-line and maintenance chemotherapy in NSCLC cases with ROS1 rearrangements. Pemetrexed showed beneficial results as first-line and maintenance therapy in four metastatic NSCLC patients with ROS1 translocations, with a PFS period of > 47 months in one patient [10]. This was considerably longer than the median PFS of 19.2 months reported for crizotinib as first-line therapy [6]. Similar results were obtained in the present case with EZR-ROS1 translocation. This patient received a pemetrexed-based regimen as front-line and maintenance therapy for 42 months overall without disease progression. These findings suggest that patients with ROS1-driven lung cancer are optimal candidates for first-line pemetrexed-based chemotherapy. A randomized control trial is needed to validate the superiority of pemetrexed over crizotinib as a first-line regimen.

The molecular mechanism underlying the prolonged PFS in ROS1-driven lung cancer treated with pemetrexed remains unknown. Low thymidylate synthase (TS) expression is positively related to the efficacy of pemetrexed in NSCLC patients and may predict a longer PFS [15, 16]. Several retrospective trials demonstrated that TS expression is significantly lower in patients harboring ROS1 translocation [17, 18]. Therefore, the favorable outcomes of pemetrexed-based treatment in ROS1-driven lung cancer could be partly attributed to low TS expression.

In the present case, NGS identified EZR-ROS1 as the oncogenic driver mutation, and crizotinib, a multi-targeted TKI, showed robust and clinically meaningful efficacy endpoints in this patient [6]. The advent of NGS revolutionized the molecular mutational spectrum of lung cancer by increasing the feasibility and range of DNA sequencing from whole genome sequencing to targeted panels, allowing the identification of targetable mutations and predicting the emergence of drug resistance [19]. NGS is capable of detecting low frequency mutations, and can screen the mutational status of different critical samples such as biopsies, cytological samples, and circulating plasma DNA, offering innovative diagnostic opportunities [20, 21]. NGS represents a highly attractive system to identify mutations and thus improve the outcome of lung cancer patients before the start of treatment [22]. An important challenge for lung cancer treatment is the spatial and temporal tumor heterogeneity, as well as clonal selection or evolution in NSCLC tumors [23, 24]. Despite the success of targeted treatment for EGFR-mutant NSCLC, the outcomes are limited by the development of TKI resistance. To dynamically monitor the histological and molecular evolution of lung cancer, secondary biopsy after each relapse combined with NGS would benefit the individualized selection of treatment regimens.

Lorlatinib is a third-generation macrocyclic ALK/ROS1 TKI with a novel chemical scaffold that shows potent antineoplastic activity against all known mutations resistant to first- and second-generation inhibitors [25–27]. The safety and efficacy of lorlatinib for ALK/ROS1-driven lung cancers were demonstrated in a phase I/II clinical trial. A recent case report described a patient who responded to lorlatinib for 8 months after becoming refractory to crizotinib [28]. The same phenomenon was observed in the present case. Therefore, we believe that lorlatinib may be the optimal choice in crizotinib-resistant cases.

CEA is a glycosylphosphatidylinositol-anchored glycoprotein that is normally produced during fetal development, and its production stops before birth [29]. High levels of CEA strongly suggest the presence of lung cancer rather than benign lung disease [30]. A rapid increase in the serum levels of CEA provides prognostic and predictive information about recurrence and mortality in NSCLC patients independent of treatment [31]. In the present case, the markedly increased serum levels of CEA were an indication of disease relapse. Therefore, routine monitoring of serum CEA levels could be beneficial in patients with lung adenocarcinoma.

In conclusion, the present ROS1-driven lung cancer case showed a positive response to pemetrexed-based chemotherapy as first-line and maintenance treatment, with a PFS of 42 months. Crizotinib and lorlatinib were used as second- and third-line therapies and both elicited a favorable response. Data from this case suggested that pemetrexed-based regimens may not be inferior to crizotinib as first-line treatment for ROS1-driven lung cancers, and lorlatinib may be an alternative treatment choice in crizotinib-resistant disease. Secondary biopsy after each relapse combined with NGS would help to dynamically monitor the histological and molecular evolution of lung cancer and may benefit the individualized selection of treatment regimens. CEA serum levels may be useful for monitoring relapse in lung adenocarcinoma.

## Abbreviations

ALK/LTK: Anaplastic lymphomakinase/lymphocyte-specific protein tyrosine kinase
ARMS: Amplification refractory mutation system
CEA: Carcinoembryonic antigen
CT: Computed tomography
EBUS-GS: Endobronchial ultrasonography with a guide sheath
FDA: Food and Drug Administration
FISH: Fluorescent in situ hybridization
INSR: Insulin receptor
NGS: Next-generation sequencing
OS: Overall survival
PCR: Polymerase chain reaction
PET: Positron emission tomography
PFS: Progression-free survival
ROS1: C-ros oncogene 1
TKIs: Tyrosine kinase inhibitors
TS: Thymidylate synthase
